# Investigating the impact of microcalcification size and volume on collagenous matrix and tissue mechanics using a tissue-engineered atherosclerotic cap model

**DOI:** 10.3389/fcvm.2025.1629285

**Published:** 2025-08-20

**Authors:** Imke L. Jansen, Deniz Șahin, Frank J. H. Gijsen, Eric Farrell, Kim van der Heiden

**Affiliations:** ^1^ Department of Biomedical Engineering, Thorax Center Erasmus MC, University Medical Center Rotterdam, Rotterdam, Netherlands; ^2^ Department of Oral and Maxillofacial Surgery, Erasmus MC, University Medical Center Rotterdam, Rotterdam, Netherlands; ^3^ Department of Biomechanical Engineering, Delft University of Technology, Delft, Netherlands

**Keywords:** atherosclerosis, human disease model, calcification, tissue engineering, mechanical testing

## Abstract

Atherosclerotic plaque rupture can lead to thrombotic cardiovascular events such as stroke and myocardial infarction. Computational models have shown that microcalcifications (calcified particles with a diameter < 50 μm) in the atherosclerotic plaque cap can increase cap tissue stresses and consequently contribute to plaque rupture. Microcalcification characteristics, such as particle size and volume fraction, have been implicated to affect cap stresses. However, the effect of these characteristics on tissue mechanics within a collagenous matrix, has not been investigated experimentally. In this study, we employ a tissue-engineered model of the atherosclerotic plaque cap with human myofibroblasts to assess the impact of microcalcification size and volume fraction on cap mechanics and rupture. To mimic human microcalcification size and volume, hydroxyapatite microparticles, in two size ranges (diameter up to 5 μm or up to 50 μm) and two volumes (1 v/v% and 5 v/v%) were incorporated homogenously throughout the tissue-engineered model. 5 v/v% of particles caused a significant lowering of the mechanical properties as was shown by a decrease in stiffness and ultimate tensile stress under uniaxial tensile loading. Additionally, the 5 v/v% of hydroxyapatite particles, in both size ranges, caused a reduced tissue compaction during culture. This might indicate that hydroxyapatite particles influence mechanobiological processes governing tissue organisation and consequent tissue mechanics. These experimental data support computational findings regarding the detrimental role of microcalcifications on cap rupture risk and highlight the importance of volume fraction. Furthermore, this study indicates an additional importance to look at the interplay between calcification, its effect on plaque cap-resident cells and the consequent effect on tissue mechanics.

## Introduction

Rupture of the fibrous cap of an atherosclerotic plaque can trigger various cardiovascular events, such as myocardial infarction and stroke (1, 2). Cap rupture is seen as a mechanical event, due to material failure of the tissue. Originally, the thickness of the fibrous cap was considered to be the primary determinant of plaque rupture. Current research however acknowledges that the heterogeneous composition of the atherosclerotic plaque plays a crucial role (3, 4). One of the components affecting this heterogeneity are microcalcifications, which by definition are calcified particles, mainly composed of hydroxyapatite (HA), smaller than 50 μm (5–7). These microcalcifications can also be found in the necrotic core of the plaque, where they can be seen as floating debris without a mechanical implication (8). Computational studies indicated that microcalcifications present in the atherosclerotic plaque cap can impact local mechanical stresses and concomitant risk of rupture (9–11). It has been suggested that microcalcifications can increase stresses within the fibrous cap by a factor of two (9, 12) to five, depending on their location (13). Several characteristics of microcalcifications can alter the increase in local stresses, such as their shape (9), size (14) and the volume they occupy (15). With regard to size, it has been suggested in computational models that microcalcifications smaller than 5 μm should not be mechanically dangerous (14). Regarding the volume fraction, it has been revealed in one computational and one experimental study that stress within the fibrous tissue increases as the volume fraction is increased (15, 16), possibly due to the increased probability of two microcalcifications being in close proximity to each other, consequently increasing local stress concentrations (9).

However, the atherosclerotic plaque is composed mainly of a heterogeneous collagenous matrix (8, 17), which has not been taken into account by computational models. These models are based on simplified cap microstructures and assume an isotropic and homogenous matrix material. Since calcification formation and collagen organisation are intertwined processes (8, 18), these two components should both be assessed in cap mechanics. Additionally, it has been suggested that hydroxyapatite particles can influence cellular behaviour by affecting cell viability (19) and collagen expression (20), highlighting a possible reciprocal relationship. To provide further insight into the effect of microcalcifications on plaque cap mechanics, systematic experimental studies are needed. *Ex vivo* material can be used for this purpose, but the large variability and effect of patient medication impedes studies of distinct cap components. As an alternative, animal models are often used, but these are limiting because they do not adequately mimic the human plaque mechanics (21). *In vitro* models can provide a variable and controllable composition to unravel the role of microcalcification characteristics in a collagenous matrix on cap mechanics. We previously developed a tissue-engineered (TE) construct to mimic the atherosclerotic cap with microcalcifications (22). In the current study we utilised this model to assess the effect of microcalcification size and volume on collagenous matrix formation and cap mechanics. In addition, we developed a cell-free collagen hydrogel model to differentiate between indirect effects of microcalcifications on cap mechanics resulting from cell-modulated effects, and direct effects due to local stress increases.

## Materials and methods

### Characterisation of microcalcification in human carotid plaques

Human carotid plaque samples were obtained from seven patients that underwent carotid endarterectomy (CEA) in the Erasmus University Medical Center Rotterdam, the Netherlands. Samples were acquired in a manner that adhered to the declaration of Helsinki and was approved by the hospital's Ethical Research Committee (MEC 2008–147). Histological cross sections of these seven CEA samples of 5 μm thickness (*n* = 90) were obtained and microcalcification location and density within the fibrous caps were analysed by Alizarin Red S staining. Additionally Scanning Electron Microscopy (SEM) was performed on one of the CEA samples.

### Culture of human vena saphena cells

Human vena saphena cells (HVSC), a type of myofibroblast, were isolated from a 72-year-old male donor after coronary bypass surgery according to previously established protocols (23). Cells were expanded in advanced DMEM (Gibco, Thermo Fisher Scientific, Breda, the Netherlands) containing 10% heat inactivated foetal bovine serum (FBS, Sigma-Aldrich, St. Louis, USA), supplemented with 50 μg/ml gentamycin (Invitrogen) and 1.5 μg/ml fungizone (Invitrogen) in a humidified environment at 37°C and 5% CO2. They were expanded until 80% confluency after which they were passaged to passage 7 for future experiments.

### Creation of tissue-engineered plaque caps with varying microcalcification composition

HA particles (CAPTAL® “R”, Plasma Biotal Limited) were used as an equivalent of microcalcifications. HA particles were previously characterised (22) and had a diameter up to 52 μm. Samples were created with HA particle size up to 5 μm (small HA, sHA) or 50 μm (Mixed HA, mHA). To create a suspension of HA particles with a diameter size up to 5 μm, a filter with a 5 μm pore-seize (pluriStrainer® 5 µm) was used. HA particles were suspended in phosphate buffered saline (PBS, Gibco, Thermo Fisher Scientific, Waltham, USA), after which the suspension was strained through the pluriStrainer®. The group with the mixed particles included small HA particles and agglomerates of these small particles that formed the bigger particles, thus creating a mixed size range. Afterwards, a 1 v/v% and 5 v/v% was created of both the mixed HA particles (diameter < 52 μm) and small HA particles (diameter < 5 μm). These volume fractions were chosen based on previous research assessing the fraction of microcalcifications in human plaque tissue (15, 22).

TE plaques were created following the methodology described previously (24). To summarize, human vena saphena cells (HVSCs) (1.5 × 10^6^ cells/ml) were seeded in 1.5 × 1.5 cm-sized fibrin gels, a suspension of bovine fibrinogen (10 mg/ml, Sigma F8630), and bovine thrombin (10 U/ml, Sigma T4648), cast between two Velcro strips (1.5 cm long). For samples with HA particles, HA was mixed with the thrombin before mixing with cells and fibrinogen, to create a total volume of 600 μm to be seeded between the Velcro strips. After seeding, the samples were cultured in a growth medium consisting of advanced DMEM (Gibco, Thermo Fisher Scientific, Breda, the Netherlands) supplemented with 10% Fetal Bovine Serum (Life Technologies), 1% Glutamax (Gibco), 0.1% gentamycin (Invitrogen), 1:167 Fungizone (Invitrogen), and L-ascorbic acid 2-phosphate (vitamin C, 0.25 mg/ml, Sigma A8960) for 21 days under static conditions (37 °C, 5% CO_2_). For the first 7 days of culture, ε-Amino Caproic Acid (ε-ACA, 1 mg/ml, Sigma) was added to prevent fibrin break-down.

### Imaging of tissue-engineered plaque caps

After 21 days, samples required for imaging (*n* = 3 per group) were rinsed with PBS. The samples with HA particles were incubated with an HA-targeting probe (IVISense Osteo 680 Fluorescent Probe, Osteosense, PerkinElmer), diluted 1:200 in PBS at 4 °C for 48 h. Following incubation, samples were rinsed with PBS and were pinned to a silicone-filled (Sylgard 184, VWR, Germany) petri-dish with sterile surgical needles. PBS was added to fully submerge the sample. A multiphoton microscope (TCS SP5 Confocal, Leica, Germany) with a Chameleon Ultra multiphoton laser (710–1,040 nm) (Coherent, USA) was used to visualize collagen architecture (25) and HA particles. Second harmonic generation (SHG) using two-photon microscopy (excitation of 880 nm) was employed to image collagen fibres in combination with confocal microscopy of the HA particles (excitation of 680 nm). Z-stacks (tile size 739 × 739 μm, step size 3 μm, pixel size 1.4 × 1.4 μm) to a depth of approximately 200 μm were collected, which is about half of the sample thickness.

For data analysis, the maximum intensity projection (MIP) of each scanned tile was obtained and further analysed using the Fiblab software (26) to extract the orientation of individual collagen fibres. Von Mises distributions were fit to the histogram of the detected collagen fiber orientations and the dispersion (*κ*) of the fibre orientations were measured per tile to assess the (an)isotropy of the engineered tissues (27). The range of *κ* is [0: 0.33], where at *κ* = 0, all collagen fibers are aligned in the main loading direction, and at *κ* = 0.33, the fibers are uniformly distributed in all possible angles.

### Mechanical characterisation: uniaxial tensile testing

Uniaxial tensile tests were performed after SHG imaging to assess the effect of HA clusters on TE caps' mechanical properties (*n* ≥ 7 per condition, including imaged samples). Before testing, samples were rinsed in PBS and imaged with a high frequency, high spatial resolution ultrasound system (VEVO 3100, FUJIFILM VisualSonics, Canada) using a linear transducer (MX550) to assess the dimensions of the central region of the sample. For uniaxial tensile testing, a custom-designed set-up (25, 28) equipped with a 20 N load cell (LCMFD-20N, Omega Engineering, USA) was used. The samples were placed in the uniaxial tensile tester, clamped at the Velcro containing portions, and the tests were performed while the samples were submerged in PBS at 37 °C. A pre-load of 0.15 N was applied to remove tissue slack and 10 cycles of preconditioning up to 10% strain were performed before the final uniaxial tensile stretching cycle until complete rupture at a strain rate of 200%/min (29). Strains and strain rate values were based on the initial clamp-to-clamp distance measured just before pre-conditioning by the actuator.

Effective tensile engineering stress–strain behaviour of the samples for the final uniaxial tensile stretch cycle was assessed, and ultimate tensile stress and strain, the tangential modulus at 5% strain, were calculated. Due to tissue compaction in the central region of the samples during culture, they developed a dog-bone shape, which is characterised by a reduction in width in the central region, as see in our previously developed model (22). This region is referred to as the reduced section and is commonly used for stress and strain assessment (30). Cross-sectional area measurements of the reduced section from the ultrasound scans were used for stress calculations and gauge length for the strain measurements.

### Tissue composition: histology

6 μm thick cryosections, cut in the z-direction, were analysed to evaluate global collagen matrix structure and HA particles. Fibrillar collagen could be visualised with Picrosirius Red (PSR, Direct Red 80, Sigma, #365548), while HA particles were stained with Von Kossa. For the PSR staining, samples were stained in 0.1% PSR solution for 60 min, after which they were washed in acetic acid and dehydrated in a series of ethanol. The von Kossa staining was performed by staining sections in a 5% silver nitrate solution (Sigma #85228) for 30 min on a lightbox. Afterward, they were rinsed in Milli-Q, counterstained with Nuclear Fast Red (Merck #1.00121.0500), and dehydrated. Stained sections were embedded in entellan (Merck #1.07961.0500) and imaged with bright-field microscopy (Olympus BX50).

Orbit Image Analysis software was used to classify the ratio of matrix to voids in Picrosirius Red stained sections (31). To be able to classify the matrix component on the slide, a classification model was trained within the Orbit Image Analysis software (31). For this classification model, six regions per image, of 3 different images were used to define the training set, to cover the entire variability of the tissue structures. Regions were manually drawn to select matrix or voids. When the model was trained on this set, a trained model was obtained, which was used to classify these components in the total data set.

### Collagen and hydroxyapatite hydrogel formulation

Neutralised collagen type I (Col I, bovine collagen, 80 mg/ml, Lifeink-200®, Advanced BioMatrix) was diluted to 55 mg/ml using sterile PBS. The Col I and PBS was mixed extensively on ice between two coupled 5 ml syringes to create a homogenous matrix. For samples with HA particles, the pluriStrainer® 5 µm was used to strain the HA particles (CAPTAL® “R”, Plasma Biotal Limited) to create the small fraction. Since the bigger agglomerates of these particles broke down into the small fraction due to shear during the mixing of the hydrogel with the HA particles in the syringes, these HA particles could not be used to represent the mixed fraction with particles up to 50 µm. Microspheres (CAPTAL® “HT” Microspheres Plasma Biotal Limited) were therefore used to mimic the mixed fraction in these experiments (msHA). The HA particles were suspended in an adjusted volume of PBS to create either 1 v/v% or 5 v/v% of particles. The hydrogel was cast in between two Velcro strips of 1.5 cm using a syringe with a sterile needle. A dog-bone shape, similar to the day 21 TE samples, was created using a silicone mould (Sylgard 184, VWR, Germany) and the hydrogel was mixed with the Velcro. The hydrogels were then transferred to a 37 °C incubator for 1 h to allow thermal fibrillation of the collagen. Afterwards, hydrogels where chemically crosslinked using N-(3-dimethylaminopropyl)-N'-ethylcarbodiimide hydrochloride (EDC, Sigma, 8.00907) and N-hydroxysuccinimide (NHS, Sigma, 130672) at a molar ratio of 5:2:1 (EDC: NHS: Collagen). The crosslinking solution was prepared by dissolving EDC and NHS in 75% acetone. Hydrogels were immersed in the crosslinking solution for 1 h at room temperature on an orbital shaker at 35 rpm to ensure uniform exposure of the hydrogels to the crosslinking agents. Afterwards, the hydrogels were washed in PBS and kept in PBS overnight for imaging and mechanical testing (*n* = 8 per condition).

### Statistical analysis

All data are presented as mea*n* ± standard deviation unless stated otherwise. Statistical analyses were performed using Prism (GraphPad, La Jolla, CA, USA). A Shapiro–Wilk test was performed for normality. In case of proved normality a One-Way Anova tests with multiple comparisons and a Bonferroni correction was done to assess differences between conditions. If there was no proven normality, a Kruskal–Wallis test was performed. For the correlation between the mechanical parameters and either cross-sectional area or quantified histological data, a linear regression model was performed and R^2^ values and *p*-values were obtained. Differences were considered statistically significant for p values < 0.05 (visualised as *p < 0.05; **p < 0.01; ***p < 0.001; ****p < 0.0001).

## Results

### Microcalcification cluster density and particle size vary in human carotid plaques

**Figure 1:**
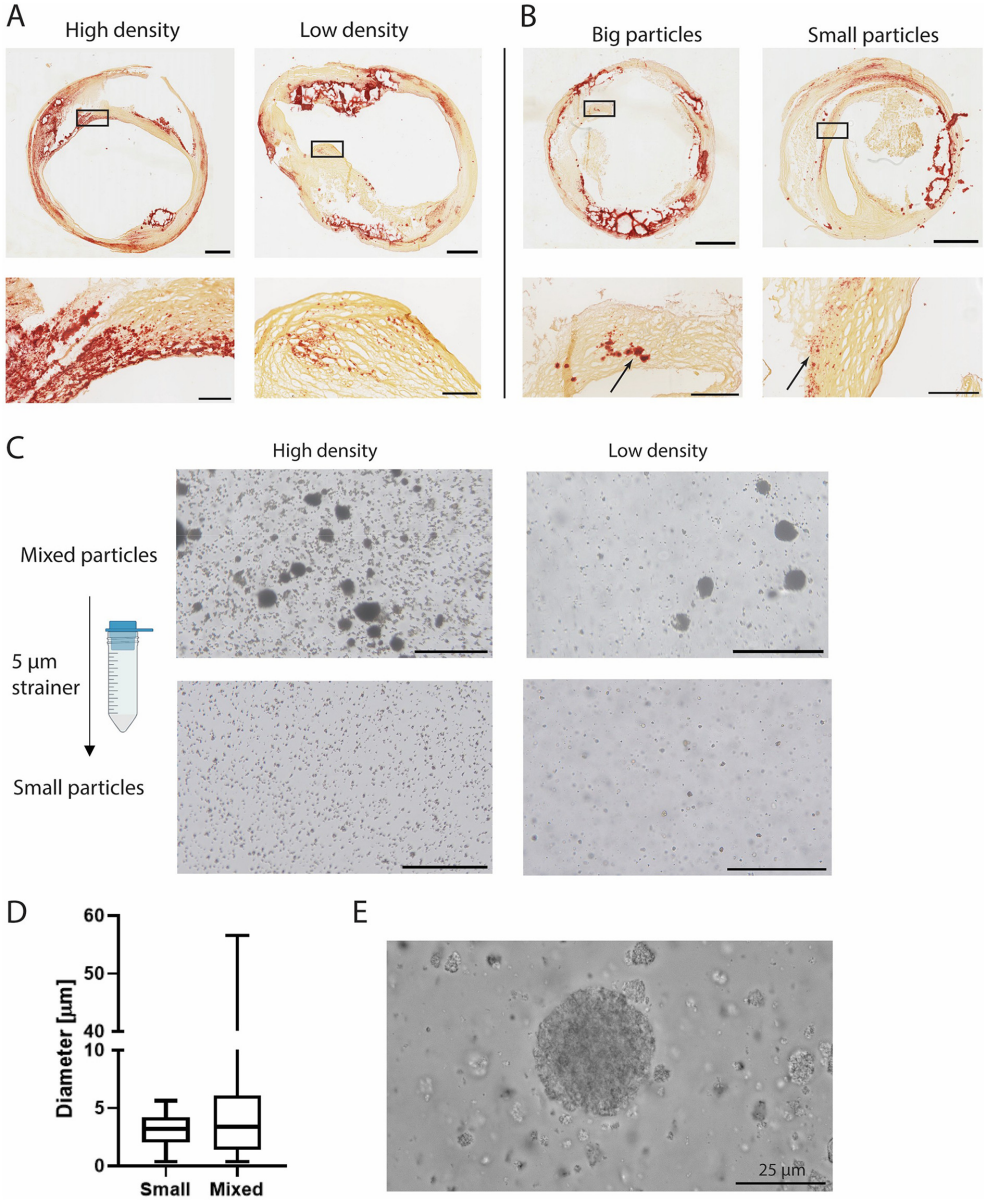
Microcalcification cluster density and particle size in carotid plaques and *in vitro*. **(A)** Representative images of carotid endarterectomy plaques (Alizarin Red staining, calcium in red) with high and low density and, **(B)** big and small particles. **(C)** Experimental conditions of HA particles with high and low density and mixed and small particles. **(D)** Quantification of HA particle size. **(E)** High magnification image of HA particles used in this study. Scalebars: **(A,B)**: Whole plaques = 1 mm, zoom = 250 μm **(C)** 200 μm **(E)** 25 μm.

Human carotid atherosclerotic plaque caps were analysed in tissue sections to assess variation in microcalcification particle size and cluster density. SEM analysis of human carotid atherosclerotic plaque tissue showed a variety in microcalcification size, shape and topography (Supplementary Figure S1A). Microcalcifications were found in both bigger, smooth topographies as well as agglomerates of smaller calcifications (Supplementary Figure S1A). The microcalcification topography which consisted out of agglomerates of multiple calcifications was mimicked by the CAPTAL® “R” HA particles in this study (Supplementary Figure S1B). Microcalcification clusters were found to have a varying density of particles in histological slides. These clusters ranged from clusters where the area on the histological slide was almost fully covered by microcalcifications, to clusters with only several microcalcifications being found within the cluster (Figure 1A). Additionally, particle size was found to differ between clusters (Figure 1B). Where some clusters contained particles up to 50 μm, other clusters only consisted of the smaller fraction of microcalcification, with sizes up to 5 μm (Figure 1B, arrows). To mimic these situations found in the atherosclerotic plaque cap, four distinct conditions were created with HA particles with either a low (1 v/v%) or high (5 v/v%) volume fraction (16) of particles and containing both small and large microcalcifications (mixed HA particles) or only the small fraction obtained by straining though a 5 µm strainer (small HA particles) (Figure 1C). The diameter in the group with small HA particles ranged up to 5.6 µm (mean 3 µm ± 1.4 µm), while this was up to 57 µm (mean 5 µm ± 5.6 µm) in the mixed HA group (Figure 1D). A high magnification image of the HA particles verified the fact that the big HA particles were an agglomeration of the smaller particles (Figure 1E). The small HA group did not include these agglomerations (Supplementary Figure S2).

### The interaction between microcalcifications and the collagenous matrix

**Figure 2:**
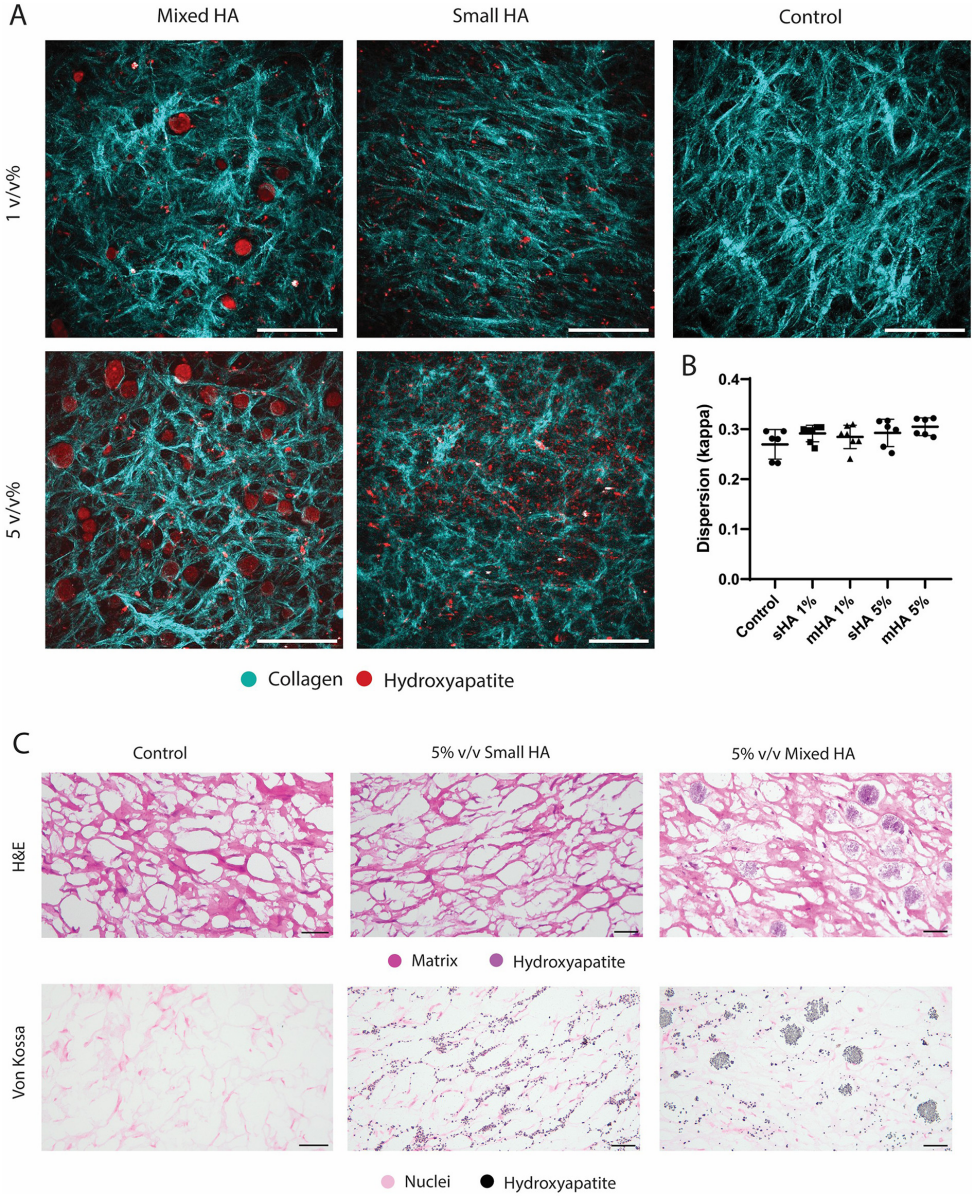
Collagenous matrix visualisation with HA particles in TE samples.**(A)** Representative SHG images of the 1 v/v%, 5 v/v% group with mixed and small hydroxyapatite particles and a control sample without HA particles. (Collagen in cyan, HA particles in red) **(B)** Dispersion quantification with dispersion coefficient *κ*. **(C)** Histological visualisation of a representative control sample, and 5 v/v% small and mixed particles with H&E (matrix and HA in purple) and Von Kossa (nuclei in pink and hydroxyapatite in black). Scale **(A)** 200 μm **(C)** 50 μm.

The tissue-engineered caps were then analysed using SHG microscopy. SHG imaging showed the HA particles and collagenous matrix formed in both control groups and all conditions with HA particles (Figure 2A). Larger HA particles could be observed within the collagenous matrix in the mixed group, while they were absent in the group with small HA particles (Figure 2A). Differences between the two volume fractions could also clearly be observed (Figure 2A). Control samples showed the formation of the collagenous matrix in the absence of HA particles (Figure 2A). The mean dispersion (*κ*) was calculated for various regions within the constructs, revealing no significant differences in collagen dispersion between conditions (Figure 2B). All groups showed a high dispersion with the means ranging from 0.27 to 0.31, indicating a highly dispersed tissue (Figure 2B).

**Figure 3:**
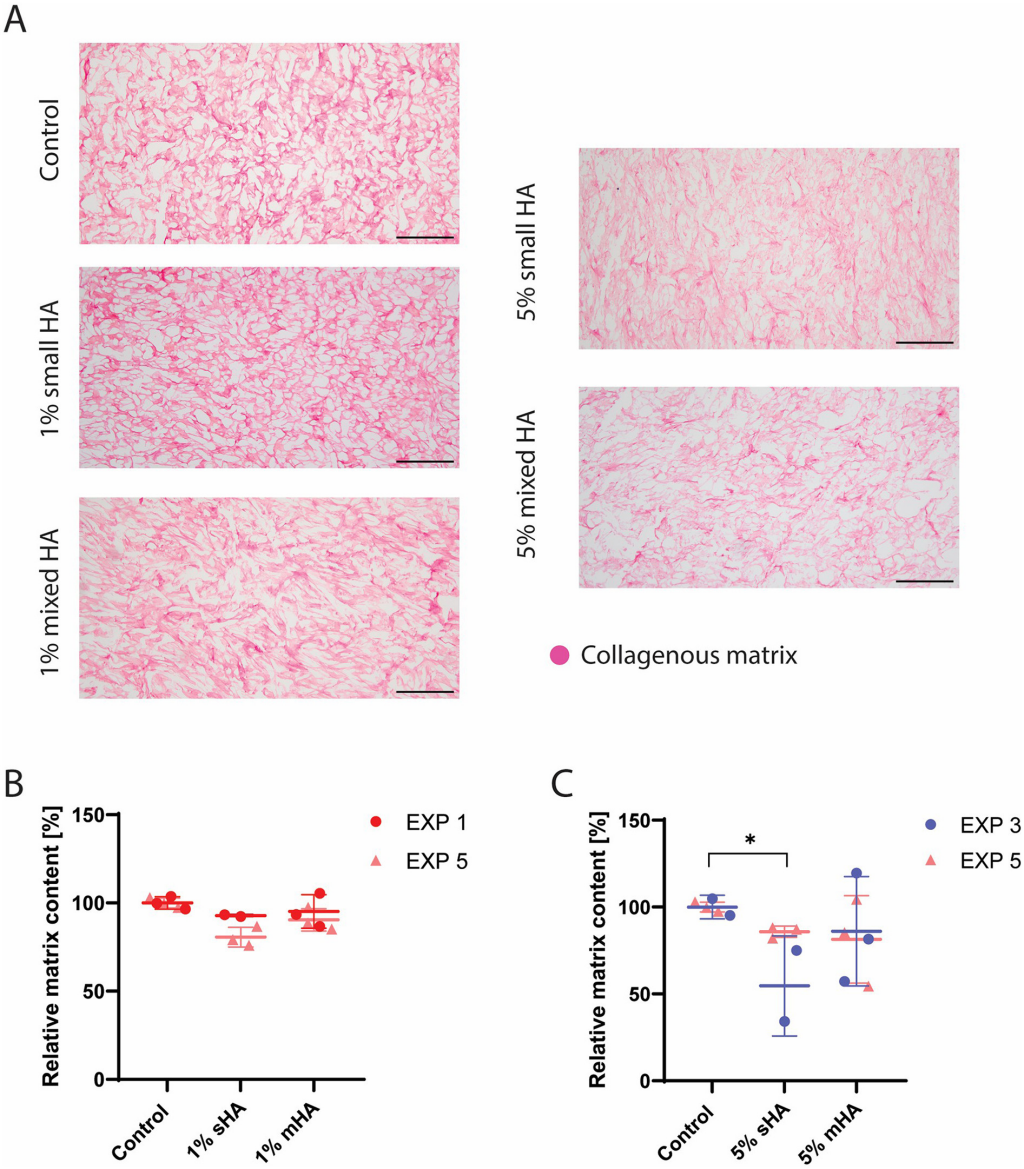
Histological analysis and quantification of all the experimental conditions. **(A)** Picrosirius Red staining showing collagenous matrix in different experimental conditions (collagenous matrix in pink). **(B)** Segmented matrix quantification of the groups with 1 v/v% HA particles using Orbit Image Analysis. ANOVA test with Bonferroni correction for multiple comparisons. **(C)** Segmented matrix quantification of the groups with 5 v/v% HA particles using Orbit Image Analysis. ANOVA test with Bonferroni correction for multiple comparisons. (B-C) EXP = individual experiments, only 2 experiments (EXP 1 and 5 for 1% groups and EXP 3 and 5 for 5% groups) per condition used for histological quantification. *n* ≥ 5 per condition. Scale **(A)** 200 μm.

Histological analysis showed that that the bigger HA particles could already be visualised with H&E staining and were found in between the collagenous matrix fibers (Figure 2C), while the smaller HA particles could not be visualised in this staining. These small particles could however be visualised in the Von Kossa staining and were found to be closely associated with the newly formed matrix (Figure 2C), instead of being present in the voids of the matrix. No calcification was observed with Von Kossa staining in the control samples. No visual differences could be observed between the matrix of the different conditions in the Picrosirius Red staining (Figure 3A). The matrix stained with Picrosirius Red of each condition was further analysed using Orbit Analysis Software by matrix segmentation (Figures 3B,C). For the groups with 1 v/v% of HA particles, no significant differences were observed with the control group (Figure 3C). However, when the concentration was increased to 5 v/v%, there was a significant decrease in the matrix content for the constructs with the small HA particles (*p* = 0.02) (Figure 3C).

### Global mechanical properties are affected by 5 v/v% HA particles

**Figure 4:**
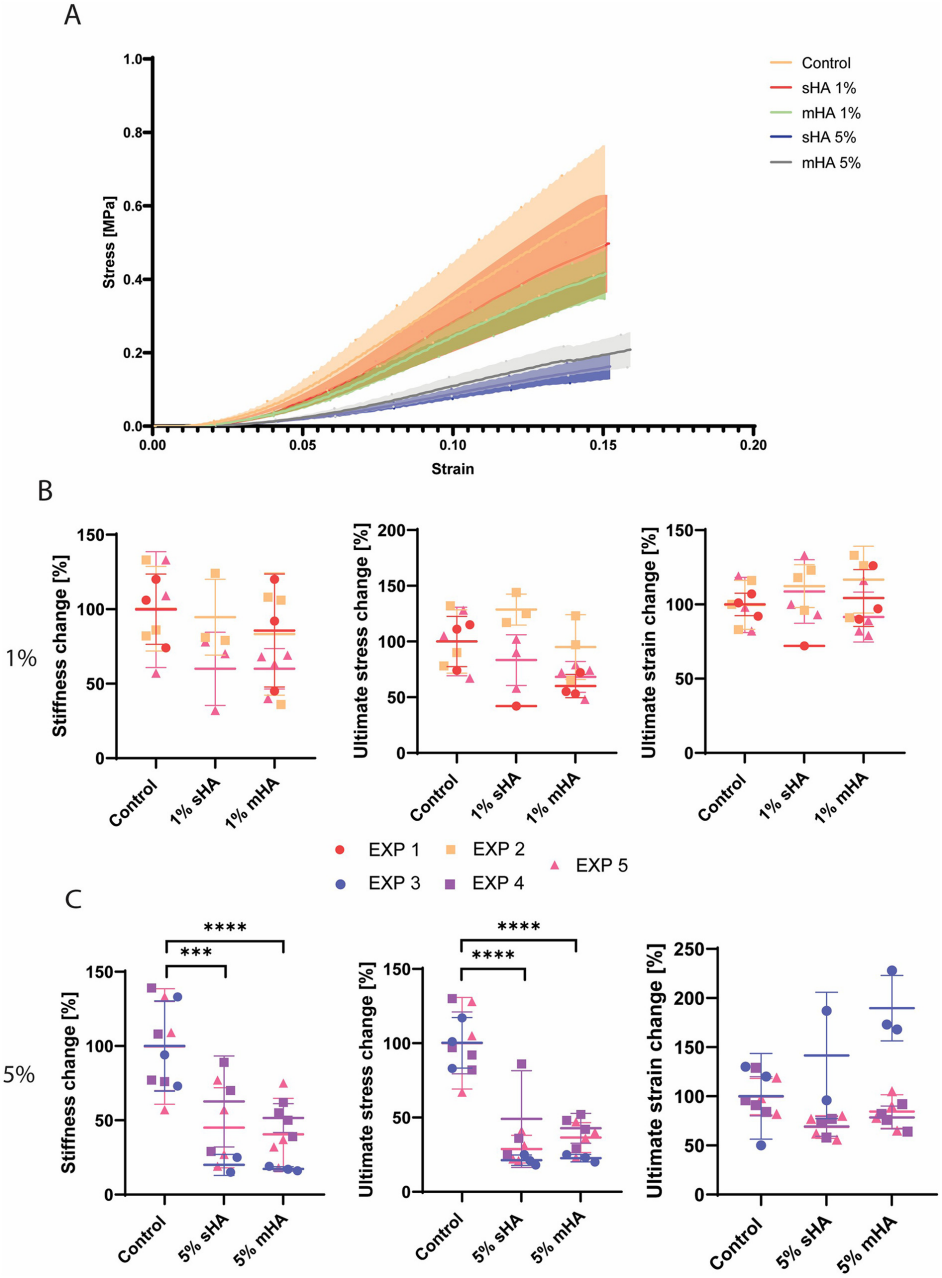
Mechanical characterisation of tissue-engineered samples **(A)** Representative mean stress-strain curves for each of the groups investigated **(B)** Normalised stiffness, ultimate stress and ultimate strain for the control and 1 v/v% sHA and mHA groups. **(C)** Normalised stiffness, ultimate stress and ultimate strain for the control and 5 v/v% sHA and mHA groups. **(B,C)** ANOVA test with Bonferroni correction for multiple comparisons. Separate experiments are shown by the different colours. EXP = individual experiment. EXP 1, 2, 5 for 1% conditions and EXP 3, 4, 5 for 5% conditions. *n* ≥ 7 per condition.

Uniaxial tensile tests revealed that all engineered constructs demonstrated a physiological strain stiffening response (Figure 4A). Constructs with 1 v/v% of HA particles visually showed little deviation from the stress-strain curve of control samples, while constructs with 5 v/v% of HA particles (both sHA and mHA) had visually diminished mechanical properties (Figure 4A). When quantifying the global mechanical parameters, stiffness at 5% strain, ultimate tensile stress and ultimate tensile strain, no significant differences were obtained between conditions with 1 v/v% of particles and control samples without HA particles (Figure 4B). The stiffness of the 1 v/v% sHA showed a decrease compared to control of 23% ± 29% (*p* = 0.25), while this was 21% ± 30% (*p* = 0.21) for the 1 v/v% mHA group (Figure 4B). For the ultimate stress this decrease was 3% ± 37% (*p* = 0.96) for the 1 v/v% sHA group and 26% ± 22% (*p* = 0.09) for the 1 v/v% mHA group (Figure 4B). No differences were found for the ultimate strain (Figure 4B).

For the groups with 5 v/v% HA particles, significant decreases in the stiffness and ultimate stress were observed (Figure 4C). The stiffness of the 5 v/v% sHA showed a decrease of 55% ± 28% (*p* = 0.0004, Figure 4C), while this was 62% ± 20% for the 5 v/v% mHA group (*p* < 0.0001, Figure 4C). For the ultimate tensile stress this decrease was 67% ± 20% for the 5 v/v% sHA group (*p* < 0.0001, Figure 4C) and 65% ± 12% for the 5 v/v% mHA group (*p* < 0.0001, Figure 4C). Again, no differences were found for the ultimate strain, possibly due to the outliers that could be identified in these groups (Figure 4C).

### The correlation between tissue characteristics and tissue mechanics

**Figure 5:**
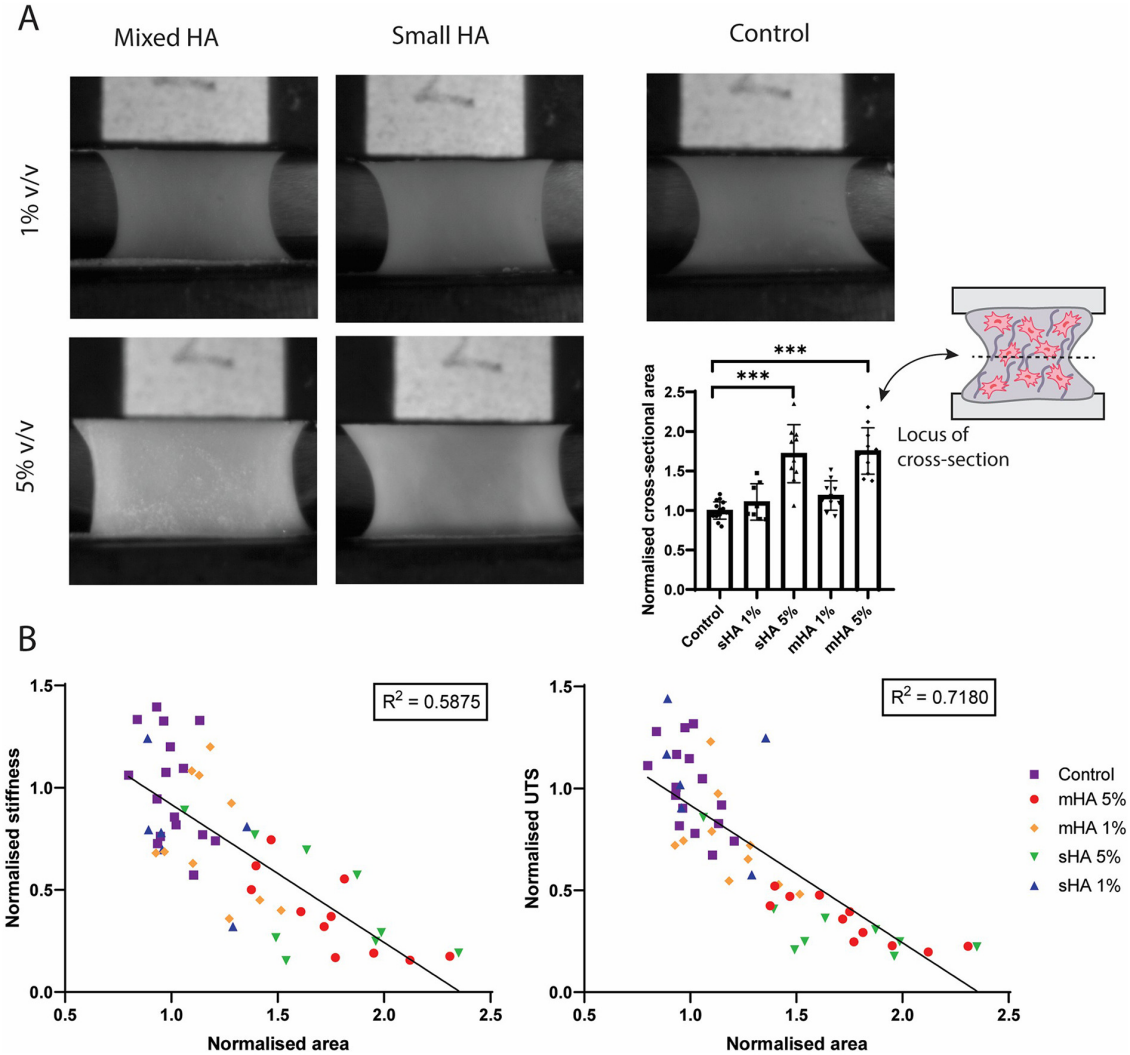
Correlation between tissue characteristics and tissue mechanics. **(A)** Representative images of the samples of each experimental condition during tensile testing and quantification of the normalised cross-sectional area obtained from ultrasound measurements. ANOVA test with Bonferroni correction for multiple comparisons. **(B)** Linear regression between normalised area and normalised stiffness and ultimate tensile stress. *n* ≥ 7 per condition.

Representative images per condition before tensile testing showed differences in compaction between groups (Figure 5A). Samples with 5 v/v% particles were visually broader compared to samples with 1 v/v% particles and control samples (Figure 5A). Ultrasound measurements made before uniaxial tensile testing, were used to quantify the cross-sectional area of individual samples and revealed differences between conditions (Figure 5A). The cross-sectional area of the group with 1 v/v% sHA and mHA were respectively 11% ± 23% and 19% ± 19% larger compared to control. For the 5 v/v% sHA this was 72% ± 37% (*p* < 0.0001) and for the 5 v/v% mHA this was 75% ± 29% (*p* < 0.0001) (Figure 5A).

The normalised cross-sectional area (measure for tissue compaction) was then correlated with the mechanical parameters stiffness and ultimate tensile stress with non-linear regression. It was found that the area inversely correlated with both stiffness (R^2^ = 0.5875, *p* < 0.0001) and ultimate tensile stress (R^2^ = 0.7180, *p* < 0.0001) (Figure 5B). The various conditions could be distinguished within these plots, with control samples being present in the higher mechanical range, while the samples with 5 v/v% particles were found in the range with diminished mechanical properties (Figure 5B). The matrix content quantified from histological analysis was also correlated with the mechanical parameters, but showed no clear correlation (Supplementary Figure S3).

### Collagen hydrogel with HA particles shows no clear effect of HA volume

**Figure 6:**
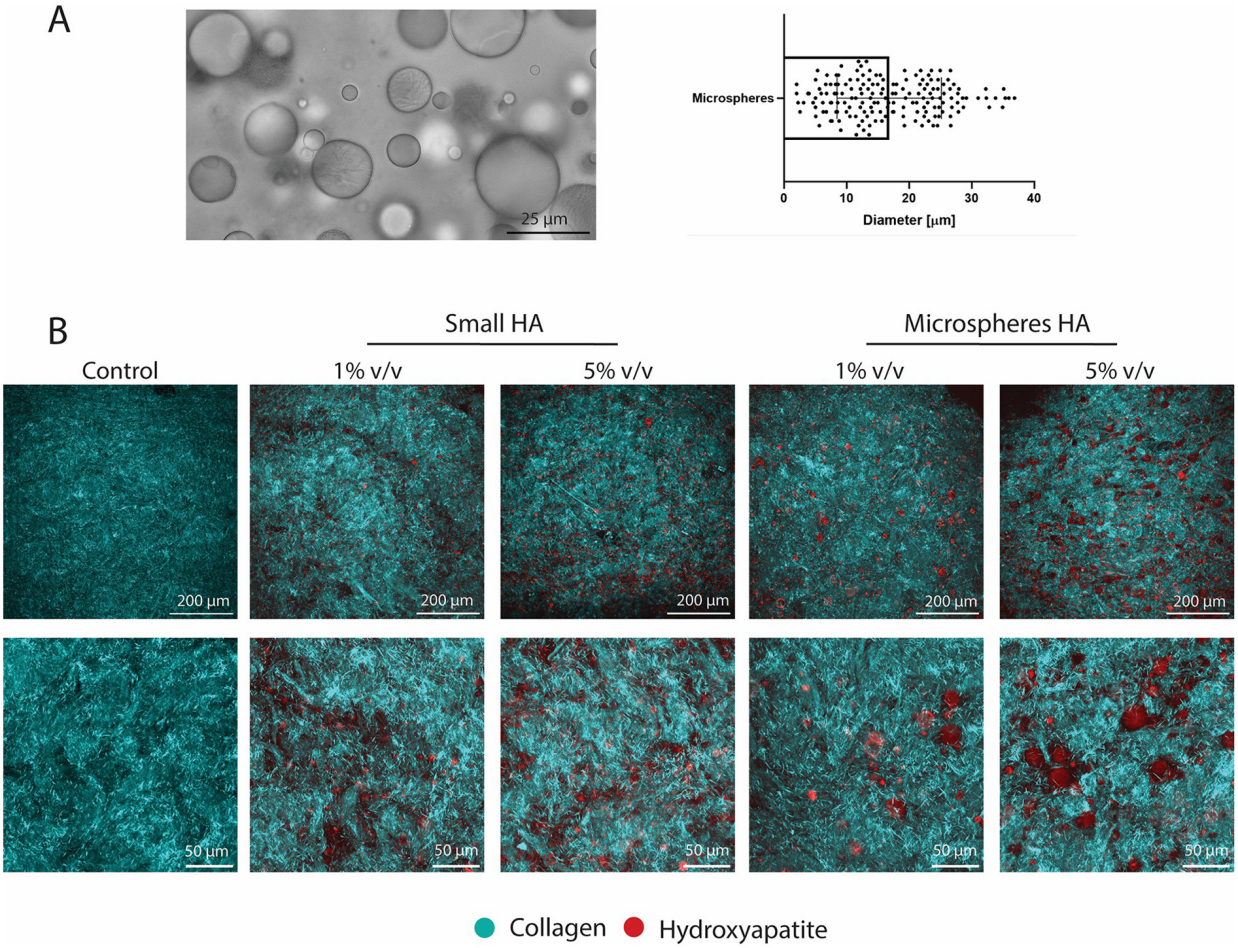
Characterisation of collagen hydrogel with hydroxyapatite particles. **(A)** Brightfield microscopy image of microspheres of hydroxyapatite as the mixed group and quantification of the diameter. **(B)** SHG imaging of all groups investigated showing collagenous matrix and hydroxyapatite particles. (Collagen in cyan and hydroxyapatite in red) Scale **(A)** 25 μm, **(B)** 200 μm (top rows) and 50 μm for zoomed images (bottom rows).

To isolate the influence of cellular activity on tissue compaction during culture on tissue compaction and consequent mechanical properties of the TE tissues, a cell-free collagen hydrogel with HA particles was created. Microspheres (CAPTAL® “HT” Microspheres Plasma Biotal Limited) were used to mimic the mixed particles in this model (msHA) (Figure 6A). Particle size was quantified and found to be up to 37 μm (mean 16.8 ± 8.3 μm) (Figure 6A). The group with small particles were the same particles as used in the TE study (Supplementary Figure S2). SHG imaging showed the collagenous matrix of the hydrogel as well as the different sizes and volumes of HA particles, with no observable differences in collagenous matrix between conditions (Figure 6B). Higher magnification images show the differences between particle size and density (Figure 6B).

**Figure 7:**
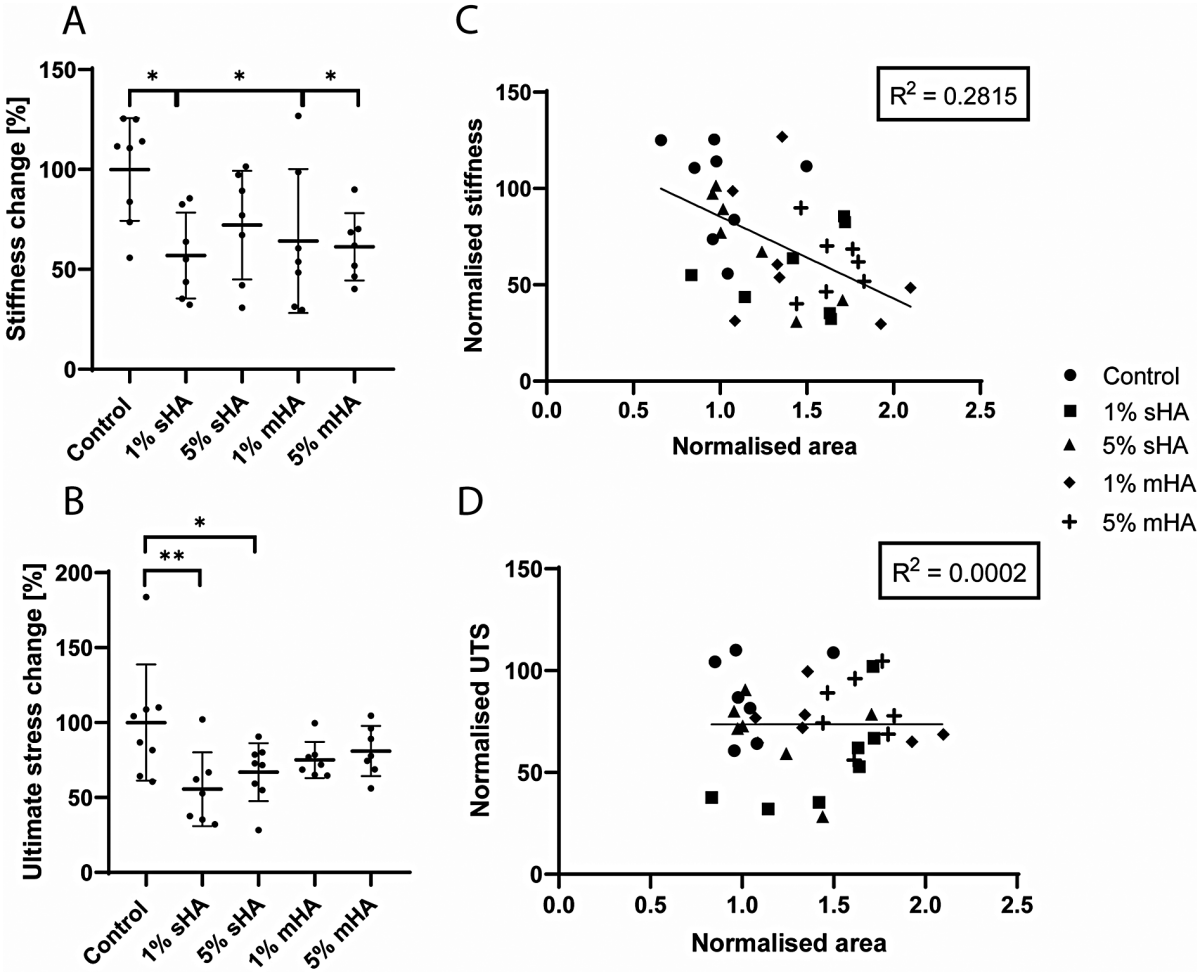
Mechanical characterisation of the cell-free collagen hydrogels. **(A)** Normalised stiffness, **(B)** Normalised ultimate stress, **(C)** Linear regression of normalised area and normalised stiffness, **(D)** Linear regression of normalised area and ultimate tensile stress. **(A-B)** ANOVA with Bonferroni correction for multiple comparisons, **(C,D)** Linear regression model. *n* ≥ 7 per condition.

Uniaxial tensile testing revealed mechanical differences between control samples and samples with HA particles (Figure 7A). For the 1 v/v% and 5 v/v% sHA the stiffness was decreased compared to control by respectively 43% ± 21% (*p* = 0.01) and 28% ± 27% (*p* = 0.15) (Figure 7A). For the 1 v/v% and 5 v/v% msHA this decrease compared to control was 36% ± 35% (*p* = 0.04) and 39% ± 17% (*p* = 0.03) (Figure 7A). The ultimate tensile stress also showed a decrease for the conditions with HA compared to control. For the 1 v/v% and 5 v/v% sHA the ultimate tensile stress was decreased compared to control by respectively 45% ± 25% (*p* = 0.005) and 33% ± 19% (*p* = 0.04) (Figure 7B). When looking at the msHA this decrease was 25% ± 12% for the 1 v/v% (*p* = 0.18) and 19% ± 16% for the 5 v/v% (*p* = 0.39) (Figure 7B).

The normalised cross-sectional area, obtained from ultrasound measurements, was correlated to the stiffness and ultimate stress values (Figure 7C–D). No clear correlation could be found for either of these parameters. For the correlation between the area and the normalised stiffness the R^2^ was 0.28 (Figure 7C), while for the ultimate tensile stress this was only 0.0002 (Figure 7D). The different experimental conditions could not be distinguished as clusters within these plots.

## Discussion

In the current study, we assessed the effect of size and volume of microcalcifications on tissue mechanics within a TE model of the atherosclerotic plaque cap with a collagenous matrix, using HA particles as microcalcification mimics. To be able to mimic clinically relevant situations, the cluster density and particle size of microcalcifications in human CEA samples was first assessed. TE samples with HA particles were created and the tensile behaviour until failure was examined, as well as possible correlations with matrix characteristics. Additionally, to rule out cell-mediated effects on mechanical properties due to interplay with HA particles, a cell-free collagen hydrogel model was developed. HA particles were shown to affect tissue compaction of cell-containing TE constructs at higher volumes, as well as lowering the global mechanical parameters. In the cell-free collagen hydrogel model, global mechanical properties were lowered independent of particle volume. These data indicate a role for microcalcifications on plaque cap biology and mechanics.

Human carotid artery samples were histologically analysed for microcalcification particle size and cluster density analysis within the human atherosclerotic plaque. In previous work, microcalcifications were discovered to be present in clusters in the atherosclerotic plaque cap with diameters ranging between 2 and 50 μm (22). In this study, we observed that both particle size and density within clusters were heterogeneously distributed. The created tissue-engineered samples contained HA particles to mimic the human microcalcifications, as well as a collagenous matrix (22), which is the main load-bearing component of the cap *in vivo* (8)*.* The HA particles were shown to be agglomerates of smaller microcalcifications, mimicking one of the topographies found *in vivo* as demonstrated in this study as well as in previous research (18)*.* Regarding the interplay between the collagenous matrix and the microcalcifications within the cap, it has been shown that the collagenous matrix can act as a scaffold for microcalcification formation (18). Additionally, calcifying extracellular vesicles have been shown to be located in the proximity of collagen fibrils (32), while bigger particles lay in between the fibers (18). In the current study, HA particles were also shown to be incorporated in the matrix dependent on their size. The small particles, with a diameter up to 5 μm, were positioned on top of the collagenous matrix, while the bigger particles (up to 50 μm), were located in the voids between the fibers. Since the collagen was produced during the culture and thus after the addition of the HA particles, this indicates that the matrix was systematically organised by the cells. This size-dependent patterning mirrors the *in vivo* situation, indicating a dynamic cellular response. However, due to the collagen formation happening after HA addition, no conclusions can be drawn on the effect of collagen on HA deposition in this study. Additionally, collagenous matrix fiber dispersion within all groups was assessed, showing a dispersed matrix for all samples, with no influence of incorporation of HA particles on this parameter. This dispersed matrix mimics a more advanced lesion, as the organisation of fiber alignment is thought to become less structured in late-stage plaques due to the biological processes involved (33). It should be noted that the dispersion is a lot higher compared to our previous study, where the collagen fibers were more aligned throughout the tissue (22). This can be due to the fact that the width of the current model was broader compared to our previous study, to optimise the dog-bone geometry for mechanical testing. This geometry might lead to increased stretching directions other than the main loading direction during culture (34), which can promote greater dispersion. Due to this increased isotropy, the relatively small changes caused by HA particles on collagen dispersion could not have been noticeable.

The reciprocal nature of the interplay between collagen and microcalcifications indicates that microcalcifications can also influence collagenous matrix organisation (8). However, research on the effect that microcalcifications have on cells and collagenous matrix formation, remodelling and organisation is scarce. In the current study, we were able to assess the effect microcalcifications might have on matrix deposition in a tissue engineering approach, as well as link this to the mechanical properties. Uniaxial tensile tests were used to assess the mechanical characteristics of our TE model. We showed that volume of the particles reduced the mechanical properties. While 1 v/v% of particles had no significant effect of mechanical parameters, 5 v/v% lowered the ultimate tensile stress and stiffness of the tissues significantly, rendering it more vulnerable to rupture. This highlights the importance of volume fraction of microcalcifications, validating a numerical study (35) and a silicone-based model (16). Furthermore, this study validates computational models on the fact that a combination of small microcalcifications and low concentrations are not mechanically dangerous (9, 14), since 1 v/v% of small particles and no effect on tissue mechanics. Controversially, in our study we show that the group with particles with a diameter up to 5 μm lower the ultimate tensile stress and stiffness of the tissues significantly at a high concentration. This is a characteristic that has not been explored specifically in computational studies so far. Our results indicate that these smaller particles might thus be harmful at higher concentrations, possibly also due to indirect effects by influencing matrix production and remodelling. High concentrations of the small HA particles reduced the collagenous matrix content within our TE samples. However, no clear correlation could be observed between the quantified matrix content and the mechanical parameters, indicating that matrix content is not the main determinant of plaque rupture. This is in line with previous studies that address that matrix content does not impact tissue mechanics significantly (36–38).

One of the other indirect effects that could play a role in tissue mechanics, is the amount of compaction of TE samples during culture. The samples with high (5 v/v%) concentrations of HA particles (both sHA and mHA) showed less compaction, and thus larger cross-sectional areas, than control samples. This indicates that both sizes of HA particles influence the amount of compaction within TE constructs, consequently influencing tissue mechanics. Furthermore, the cross-sectional area of samples correlated negatively with both stiffness and ultimate tensile stress, suggesting that more compacted samples could resist higher stresses. This is in line with previous literature of vascular constructs with aortic smooth muscle cells in 3D matrices (39), where increased hydrogel compaction led to a denser matrix and improved mechanical properties. The current study shows for the first time that microcalcifications influence compaction and consequently mechanical properties. This could be linked to collagen fiber remodelling *in vivo*, as limited compaction, possibly due to microcalcifications, decreases integrity of the plaque cap, making it less stable and prone to rupture (40). For future studies, samples with a high inter-sample heterogeneity could be created to study the effect on the mechanical response. In the current study, various outliers could be detected, most probably due to biological variation, and future work could focus on the analysis of individual tissue properties of these samples and the link to their mechanical properties.

To eliminate the effect of HA particles on HVSC mediated collagen deposition and remodelling and the consequent effect on tissue mechanics, an additional cell-free collagen hydrogel model was developed. The advantages of cell-free hydrogel models (41, 42) include that they are able to mimic composition and structure, with modifiable mechanical properties. Furthermore, they can be created without the necessity of cells, thus being a lot more time-efficient and eliminating effects these cells might cause in TE models, such as differences in compaction and matrix production. When assessing the mechanical properties of the collagen gels, the decrease in stiffness and ultimate tensile stress due to the incorporation of HA particles compared to control samples was shown. In the collagen hydrogel, HA particles caused an approximate decrease of 36% in stiffness compared to control and 30% for the ultimate tensile stress. This indicates that the effect of microcalcifications on tissue mechanics cannot only be attributed to the effect they have on cells and consequent tissue remodelling, as the cells are not present in the hydrogel model. The collagen hydrogel model shows that microcalcifications also directly influence tissue mechanics, possibly due to local stress accumulations (43). However, the observed decrease in mechanical properties was stronger in the TE model, with decreases up to 75% for both stiffness and ultimate stress. Both the direct effect observed in the collagen hydrogel model and the indirect effect resulting from the interplay with cells in the TE model could thus affect atherosclerotic plaque mechanics.

While the global effect of HA particles on tissue mechanics was shown in the collagen hydrogel model, no statistical differences could be observed between the various HA conditions. The decrease in mechanical parameters seemed to be independent of both volume and size. Various hypotheses to why no differences were observed between various volumes of HA particles in the collagen hydrogel have to do with the differences between the TE model and the hydrogel model. Firstly, the collagenous matrix in the hydrogel lacks complexity that can be seen in TE model (44), such as the inclusion of glycosaminoglycans, as was demonstrated previously to be present in our TE samples (22, 24). Secondly, there can be differences in crosslinking of the matrix (45), affecting the impact that microcalcifications have within this matrix. In the TE model, enzymatic lysyl oxidase (LOX) is responsible for the crosslinking of the matrix, which can be achieved in a three-week culture period (24). For the collagen hydrogels, EDC/NHS was used as a crosslinker, which creates chemical crosslinks by amide bonds (46). EDC/NHS crosslinking causes a reduced elasticity of the collagen matrix, while LOX crosslinking often has a minimal effect on tissue elasticity (47). The difference in crosslinking create varying tissue properties, which can influence the stress distribution of the tissue (43). These altered material properties have been shown computationally to influence the stress amplification that microcalcifications can cause (43). Furthermore, it was shown that 1 v/v% of particles affected the collagen hydrogel significantly compared to the non-significant effect measured in the TE model. This might be explained by the collagen model being more simplistic and therefore more susceptible to the incorporation of microcalcifications, whereas the TE cap model might be able to adapt to the incorporation of this low concentration of HA particles. The lack of a further decrease in mechanical properties with a 5 v/v% concentration of HA particles could be due to saturation. The threshold for rupture might have been reached already at a concentration of 1%, or even lower. Even a small amount will significantly interfere with the simplistic matrix and is not amplified with more particles, unlike the TE model with a more complex and adaptable matrix. The hydrogel model can therefore potentially be seen as a less dynamic collagenous construct compared to the matrix of the TE model. This highlights that differences in the matrix could affect the influence of microcalcifications on tissue mechanics and emphasises the benefits of using a TE model.

A limitation of this study, is the relative simplicity of the created samples when compared to the complexity of the atherosclerotic plaque cap. To be able to assess the effect of size and volume fraction of microcalcifications, the particles were homogeneously incorporated into the tissues, while it is known that they are present in clusters and not homogeneous throughout the atherosclerotic plaque cap (22). We have previously developed a model with microcalcification clusters (22) and this model was used to further recapitulate and study more clinically relevant situations, such as various localisations of microcalcifications within the atherosclerotic plaque cap (48). Additionally, multiple topographies and shapes of microcalcifications could be analysed. Furthermore, the effect of microcalcifications within more anisotropic collagenous matrices could be assessed in both the TE model as in the hydrogel. We previously showed that microcalcifications can influence collagen dispersion in a more aligned collagenous matrix, which could be a reason for altered mechanical properties. The effect of volume and particle size could be assessed in this aligned collagenous TE model. Additionally, by applying strain to the collagen hydrogel while polymerising, a more aligned matrix can be created (47), to also test these characteristics in the cell-free model. Moreover, in future studies other cap components could be added to further elucidate the role between various cap components and their interplay with mechanics. Additionally, the model could be utilised to focus on biological processes responsible for the changes in matrix production and tissue compaction in the presence of HA particles. We speculate that the smallest HA particles could be internalised by the cells, causing altered collagen expression (20). Additionally, mechanical stretching of the cells overlying the HA particles could play a role (49), which could be analysed by traction force microscopy (50). Furthermore, cellular phenotype could be assessed, as myofibroblasts could be transdifferentiating and consequently lose their contractile properties in a calcified environment (51), leading to diminished tissue compaction. This research could further elucidate the effect of HA particles on cellular mechanisms and collagenous matrix formation and what this might indicate for the *in vivo* situation.

## Conclusion

With a tissue-engineered model of the atherosclerotic plaque cap with a collagenous matrix and microcalcifications the effect of microcalcification particle size and volume fraction on tissue mechanics was assessed. We confirmed the detrimental role of microcalcifications in a cell-free collagen hydrogel model, with a decrease in tissue stiffness and ultimate tensile stress compared to control. Microcalcifications in both size ranges (<5 μm and < 50 μm) were shown to influence tissue compaction in the TE model at high concentrations. The microcalcifications caused a decrease in mechanical properties, which correlated with tissue compaction. This indicates an additional importance to assess the interplay between microcalcifictions, cap-resident cells and their extracellular matrix. The effect of microcalcifications on tissue mechanics is thus not exclusively due to their direct effect on local stresses, but also indirect due to their interplay with cells and their collagenous matrix production and remodelling. The reciprocal relationship between calcifications and cells leads to a stronger decrease in mechanical strength of the cap, consequently leading to a more rupture-prone cap.

## Data Availability

The original contributions presented in the study are included in the article/Supplementary Material, further inquiries can be directed to the corresponding author/s.
