# Supplementary material for: Investigating the impact of microcalcification size and volume on collagenous matrix and tissue mechanics using a tissue-engineered atherosclerotic cap model
Source: Front Cardiovasc Med. 2025 Aug 20;12:1629285. doi: 10.3389/fcvm.2025.1629285 (PMC12405381; doi:10.3389/fcvm.2025.1629285)
Supplement: Supplementary file 2 [file Datasheet2.docx]

**Supplementary file:** **Investigating the impact of microcalcification size and volume on collagenous matrix and tissue mechanics using a tissue-engineered atherosclerotic cap model**

**
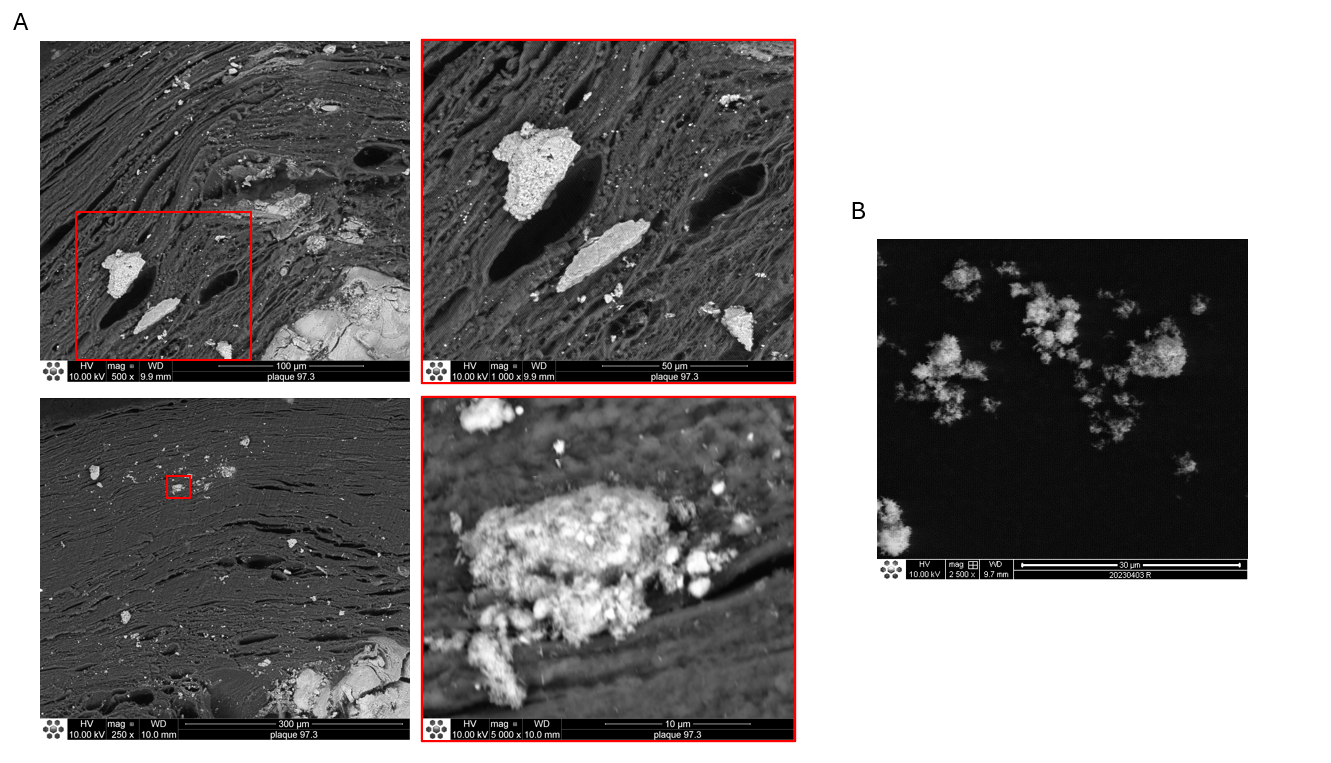
**

Supp. Fig 1: Scanning Electron microscopy images of human carotid endarterectomy samples (A) as well as the hydroxyapatite particles used in this study (B).


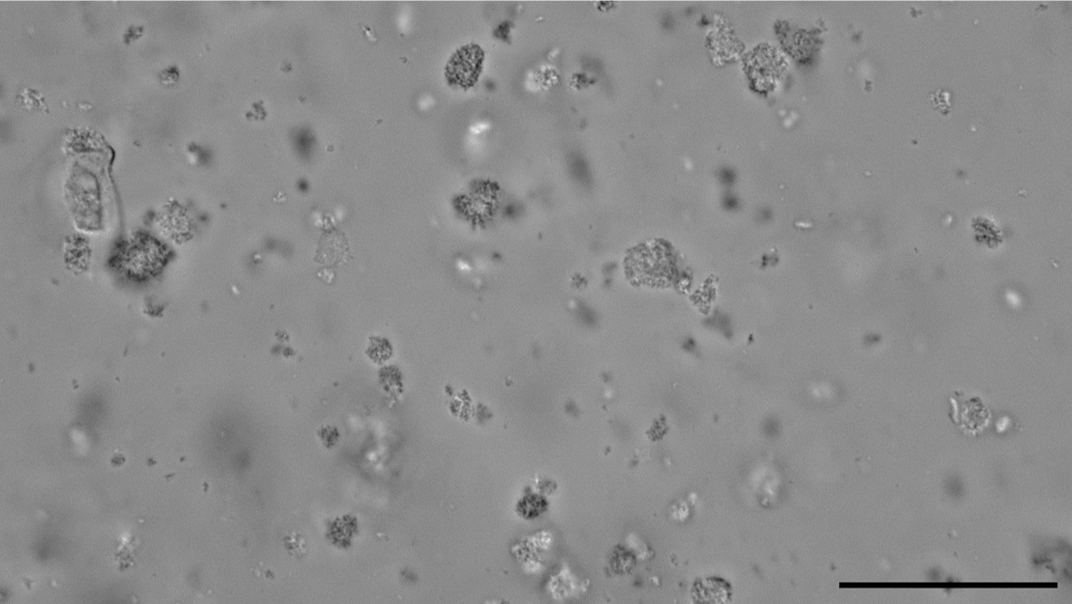


Supp. Fig 2: Brightfield image of small HA particles. Scale = 25 μm


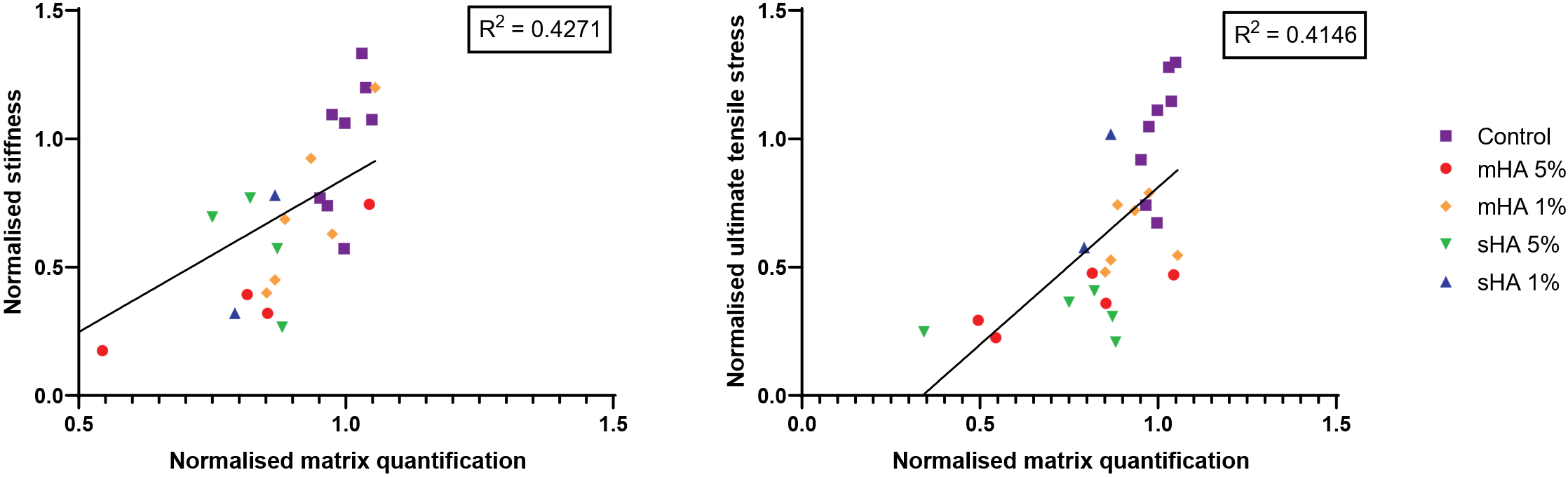


Supplementary figure 3: Linear regression of normalised matrix quantification and normalised stiffness and tensile stress.
